# 100. "They Never Asked Me": A Mixed-Methods Study to Evaluate the *INJECT-RESPECT* Tool for Assessing Infectious and Non-Infectious risks of Injection Drug Use

**DOI:** 10.1093/ofid/ofad500.016

**Published:** 2023-11-27

**Authors:** Edward C Traver, Sarah Schmalzle, Meghan Derenoncourt, Onyinyechi Ogbumbadiugha-Weekes, Habib Omari, Shivakumar Narayanan, Christopher Welsh, Sarah Kattakuzhy

**Affiliations:** University of Maryland School of Medicine, Baltimore, MD; University of Maryland School of Medicine, Baltimore, MD; University of Maryland, Baltimore, Baltimore, Maryland; Institute of Human Virology, University of Maryland School of Medicine, Baltimore, Maryland; University of Maryland Baltimore, Baltimore, Maryland; University of Maryland School of Medicine, Baltimore, MD; University of Maryland School of Medicine, Baltimore, MD; Institute for Human Virology (IHV), University of Maryland School of Medicine, Baltimore, Maryland

## Abstract

**Background:**

Injection drug use (IDU) increases risk for myriad health complications, including infections (e.g., HIV, cellulitis, and endocarditis), tissue injury, and overdose. Despite the prevalence of IDU and associated harms, tools to assess risk and provide harm reduction counseling are uncommon. We sought to create and refine a tool for assessment of IDU practices, using the input of experienced clinicians and people with lived experience of IDU.

**Figure 1.**
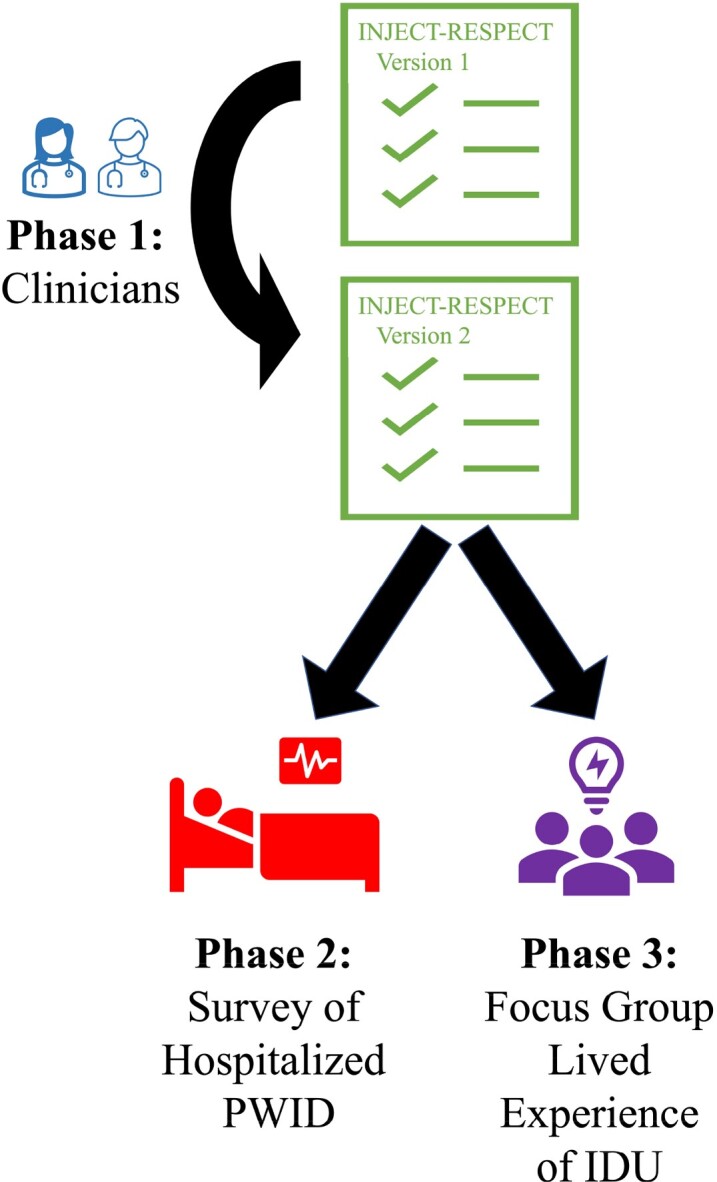
Study Design.

The INJECT-RESPECT tool was evaluated and refined in 3 phases, each seeking input from different groups of stakeholders. IDU, injection drug use; PWID, people who inject drugs.

**Methods:**

A 3-part process of feedback and refinement was used (Figure 1). The authors developed *INJECT-RESPECT* version 1 based on existing literature and their experience. In Phase 1, 12 attending physicians in 6 specialties provided feedback on *INJECT-RESPECT* version 1, which was incorporated into version 2. In Phase 2, 20 people who were hospitalized with infectious complications of IDU were interviewed about *INJECT-RESPECT* version 2 and asked (*1) Were you asked this question?* and *(2) Is this important to ask?* Responses were recorded as “Yes”/“No”. In Phase 3, a focus group was conducted with 7 people with lived experience of IDU, focused on healthcare providers’ approach to discussing IDU practices.

**Results:**

Of the 20 hospitalized participants in Phase 2, 45% were women and the median age was 37 years (range 24-48 years). Only 7 questions (11%) were asked of most respondents, yet 29 questions (44%) were classified as important by ≥75% of respondents (Table 1). Importance was correlated with whether the question was asked (Spearman correlation coefficient 0.71; *p* < 0.001; Figure 2). In the focus group, important themes were shame, stigma, and trauma (Figure 3).

**Table 1.**
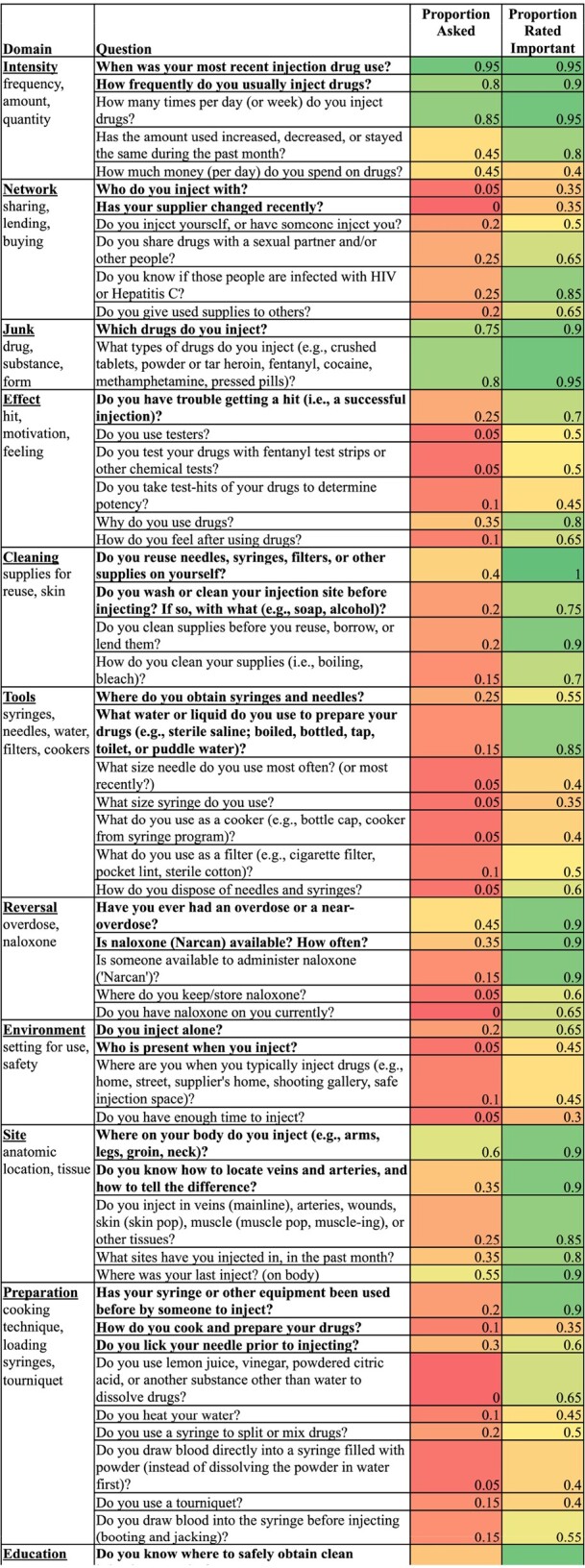
INJECT-RESPECT tool Version 2 and Responses from Hospitalized People who Inject Drugs

The INJECT-RESPECT tool Version 2 for assessing IDU-related risks is divided into 13 domains. Bolded questions are designed for priority use, such as a time-limited clinical setting. The proportion of respondents (n = 20) who were asked the question and who thought it was important are reported. The proportions are colored on a spectrum from red (0.0) to yellow (0.5) to green (1.0).

**Figure 2.**
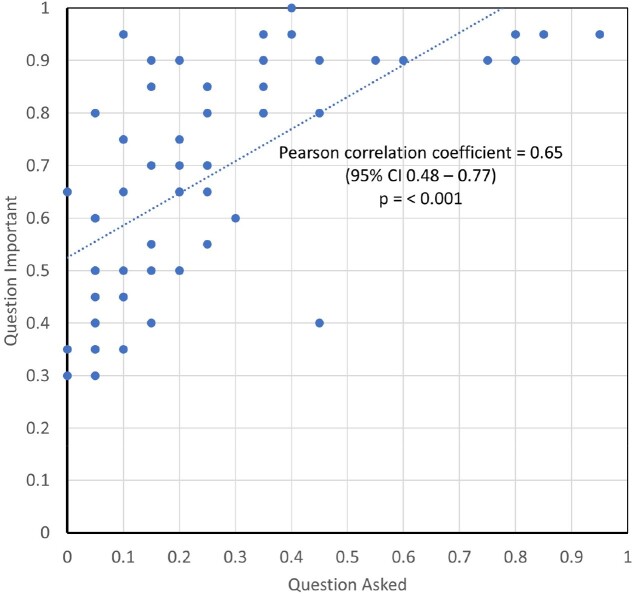
Correlation between Question Asked and Question Importance.

The importance of the INJECT-RESPECT questions—as assessed by hospitalized people who inject drugs in Phase 2—was positively correlated with whether the participants had been asked the questions. (Spearman correlation coefficient 0.71; 95% CI 0.48 - 0.77; p < 0.001.)

**Figure 3.**
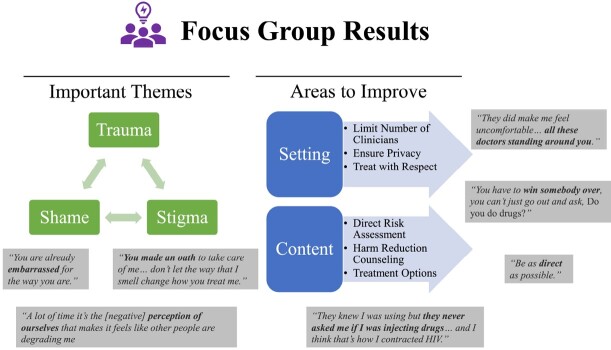
Focus Group Results

**Conclusion:**

People admitted with acute complications of IDU were not asked questions about their IDU practices that they and surveyed clinicians deemed important. Our results suggest more important questions are asked more frequently, but many important questions are rarely asked. One third of questions were rated as not important by ≥50% of respondents; these questions will be refined or removed. The focus group yielded important considerations for discussing IDU. The feedback from Phases 1, 2, and 3 will be used to further improve the INJECT-RESPECT tool. Future efforts will focus on implementing the tool with clinicians and evaluating its real-world use (Figure 4).

**Figure 4.**
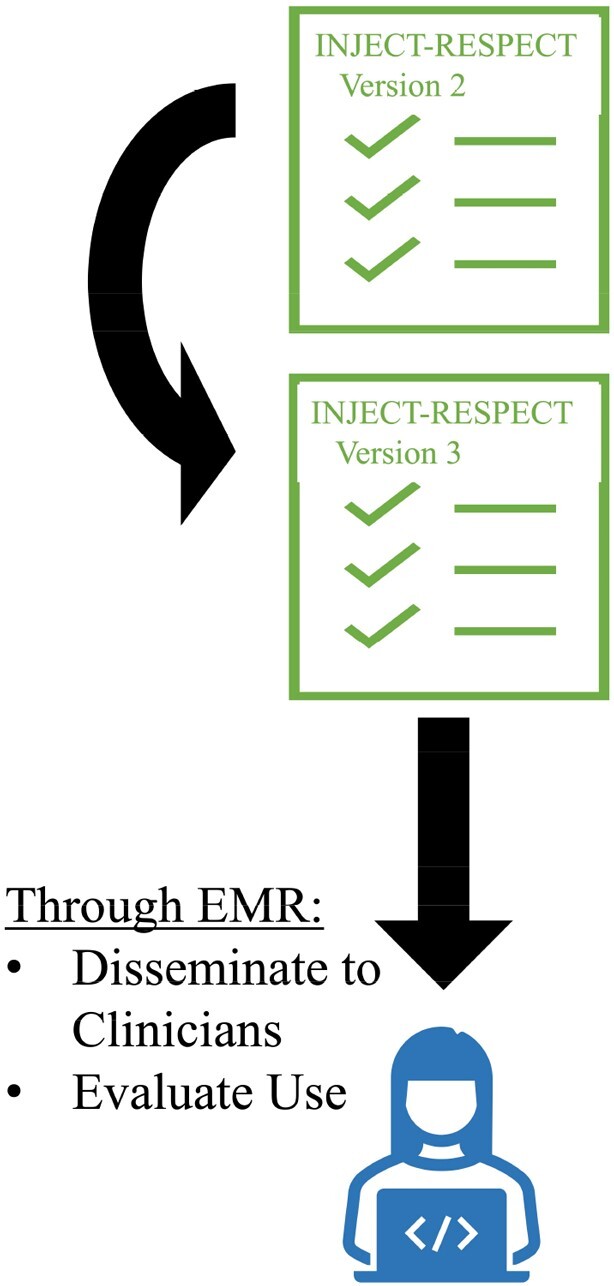
Future Plans for INJECT-RESPECT

INJECT-RESPECT will be refined based on the results of this study. It will be disseminated to clinicians through the EMR, which will also allow evaluation of how it is used. EMR, electronic medical record.

**Disclosures:**

**All Authors**: No reported disclosures

